# Interrater reliability and experiences of Assessment, Intervention, and Moving-on 3 Assessment Model in a multidisciplinary Norwegian sample

**DOI:** 10.3389/fpsyg.2022.1019739

**Published:** 2022-12-05

**Authors:** Monica Jensen, Ingunn Rangul Askeland, Ragnhild Bjørknes

**Affiliations:** ^1^Betanien Hospital, Bergen, Norway; ^2^Faculty of Psychology, University of Bergen, Bergen, Norway; ^3^Alternative to Violence (ATV), Oslo, Norway; ^4^The Norwegian Center for Child Behavioral Development, Oslo, Norway; ^5^Department of Health Promotion and Development, Faculty of Psychology, University of Bergen, Bergen, Norway

**Keywords:** harmful sexual behavior, adolescents, AIM3 assessment, interrater reliability, Norwegian context

## Abstract

**Background:**

Assessing minors with harmful sexual behavior (HSB) is a complex and sensitive task. The AIM3 Assessment Model was developed to assist practitioners with information collection and HSB evaluations.

**Objective:**

In this study, we explore the interrater reliability and the practitioners’ experience with the AIM3.

**Participants and setting:**

The multidisciplinary sample (*n* = 56) was recruited in Norway. The participants’ mean age is 43.2 years (SD 9.5). The sample is 79% female and 21% male. Mean years of experience is 17.6 years (SD 9.5).

**Methods:**

The participants used the Norwegian version of the AIM3 to score three case vignettes. A survey containing questions about competence and experience was filled out. We used the intraclass correlation coefficient (ICC) to estimate interrater reliability as well as descriptive statistics to show experience.

**Results:**

The estimated ICC for overall AIM3 factors is 0.547 (95% CI = 0.471, 0.634); for domain scores, the estimated ICC is 0.697 (95% CI = 0.548, 0.852). Both are in the moderate range. The majority of the participants reported that they will probably use the AIM3 in the future and that their experience with the AIM3 was highly useful with, for example, empirically informed decision-making and for intervention and safety planning.

**Conclusion:**

The moderate ICC results and the sample’s generally positive experience with the AIM3 may indicate further usefulness in a Norwegian multidisciplinary setting. We provide recommendations on how the AIM3 and similar HSB assessments can be further evaluated and developed.

## Assessment of harmful sexual behavior

In Western societies, estimates suggest that 30%–50% of child sexual abuse involves other children or adolescents who have displayed harmful sexual behavior (HSB; Barbaree and Marshall, 2006; Finkelhor et al., 2009; Hackett, 2014). The field of work centered on these minors requires professional confidence and competence. Therefore, the development of reliable assessment tools is greatly needed to assess consistently, and with less personal bias, adolescents who have displayed HSB, enhancing the possibility of reducing the influence of personal attitudes and values on professional judgments and decisions. Research has also facilitated the use of assessment and decision-making processes that are more structured in preference to solitary clinical intuition and judgments (Ægisdóttir et al., 2006; Smid et al., 2013; Munro, 2020; Douglas and Otto, 2021). The Assessment, Intervention, Moving-On 3 (AIM3) Assessment Model is a structured framework that assists multidisciplinary practitioners in assessing HSB within the context of their family and their environment (Leonard and Hackett, 2019a, 2021). In this study, we investigate the interrater reliability (IRR) of judgments made by Norwegian practitioners and their experiences using this tool.

The practitioners’ assessment, evaluation, and subsequent recommendations of interventions and safety restrictions will often have a major influence on the immediate and future rights and lives of minors and their families—both for the young persons who have displayed HSB and for their victims (Smid et al., 2013). Thus, the practitioners should be aware of the HSB tools’ varying strengths and limitations, psychometrics, and the settings and purpose of the screening or assessment tool before using them (e.g., forensic and correctional settings vs. community, outpatient and clinical settings, predictive vs. informal objectives; *cf.* Evers and Sjöberg, 2013; Muñiz et al., 2013; Douglas and Otto, 2021).

Over the last two decades, assessment tools have developed in this area (Print et al., 2009; Miccio-Fonseca, 2013; Leonard and Hackett, 2019a; Prentky et al., 2020). The field has moved from unguided clinical judgment to actuarial prediction of recidivism risk based on historical and static factors. Today, the field focuses on assessing potentially changeable dynamic and protective factors, and further evaluation and development are ongoing (Griffin and Beech, 2004; Hempel et al., 2013; Craig and Rettenberger, 2016; Miccio-Fonseca and Rasmussen, 2018). Empirical research has found existing HSB recidivism risk prediction tools to be inaccurate, thereby inducing the development of HSB assessment tools informing the intervention and safety planning (e.g., Viljoen et al., 2012; Hempel et al., 2013; Barra et al., 2018). Researchers have become increasingly aware that such assessment tools must account for the rapid physical and psychological development, social expansion, and change of childhood (Dahl et al., 2018) and the emergent use of the internet and social media (Belton and Jackson-Hollis, 2016).

Last, the development and objectives of assessment tools have been driven by a recognition of the minor HSB population’s heterogeneous backgrounds, characteristics, and pathways regarding HSB (Knight and Prentky, 1993; Andrade et al., 2005; Finkelhor et al., 2009; Hackett, 2014; Jensen et al., 2020). This heterogeneity implies that initial assessment tools need to be broad in both assessing and distinguishing individual characteristics. Situational and ecological factors that can eliminate, reduce, or modify risk factors or promote protective factors and well-being must be accounted for (Beech and Ward, 2004; Griffin et al., 2008; Allardyce and Yates, 2017).

### The Assessment, Intervention, Moving-on 3 Assessment Model

The AIM3 is an evidence-informed assessment tool designed to provide practitioners with a structured, holistic, ecological framework for collecting information on and evaluating HSB by identifying both sexual and nonsexual needs (Leonard and Hackett, 2019a, 2021). This tool was developed primarily for use in outpatient and community settings by multidisciplinary practitioners assessing young people aged 12–18 years. The AIM3 has five domains: Sexual Behavior, Nonsexual Behavior, Developmental, Environmental/Family, and Self-Regulation. The AIM3 is a dynamic assessment framework designed to be used both in the initial stages and in the evaluation of the young person’s development during interventions. Using the AIM3 terms, the practitioners must rate or assess their concerns on 25 AIM3 factors within the five domains. Seventy-one suggested items are proposed to guide these factors’ analysis.

The AIM3 has a standardized scoring and analysis sheet for each domain and factor. The framework measures both historical and current factors. The historical factors are important in the AIM3, because they influence the current presentation and functioning of the young person. Practitioners plot the summarized scores for each domain on a profile graph, indicating which concerns must be managed immediately and which are more moderate or long-term, as well as whether any areas of relative strength are present and can be built upon.

The AIM3 is designed to connect, when relevant, to the AIM & National Society for the Prevention of Cruelty to Children (NSPCC)—Technology-Assisted HSB Guidance (Allotey and Swann, 2019a, 2019b) and the AIM Intervention Guidance, Second Edition (Guilhermino and McCarlie, 2019a, 2019b).

Internationally, there are not currently any available or published studies or reports on the development, evaluation, or psychometrics of the AIM3 assessment model. In this exploratory study, we will estimate IRR by examining how Norwegian multidisciplinary practitioners rate three case vignettes shortly after formal AIM3 training.

### Interrater reliability

Hallgren (2012) described IRR assessment as a way of quantifying the degree of agreement between two or more raters who independently rate a feature of a set of subjects (e.g., people, things, or events). The intraclass correlation coefficient (ICC) is one of the most commonly used IRR indices for ordinal, interval, and ratio variables (Hallgren, 2012; Landers, 2015; Hanson, 2022).

Traditionally, many ICC studies in the literature have used only two raters, often highly motivated and with interchangeable characteristics, to estimate the IRR. In contrast, we want to estimate the IRR for the use of a specific assessment tool used by various multidisciplinary practitioners. The current study’s design, using multiple trained “real-life” raters, is inspired by studies conducted by Hanssen-Bauer et al. (2007), Sutherland (2010), Sutherland et al. (2012), and Webster et al. (2006).

### The Norwegian multidisciplinary context

Norway has a population of about 5,400,000. About 21% are children under the age of majority (18). The age of sexual consent is 16, and the age of criminal responsibility is 15. In the last decade, there has been an increasing public, professional, and governmental awareness of children and adolescents who have displayed problematic behaviors and/or HSB (Holt et al., 2016; Askeland et al., 2017; Health and Care, 2017; Vorland et al., 2018).

Historically, disclosure, assessment, and interventions for minors having displayed problematic behaviors or HSB have been unsystematic. Interventions have mainly been treatment-oriented and delivered through public health and social outpatient services (e.g., in Norway, the state agency Children Welfare Services or the Children and Adolescent Mental Health System). Since 2005, some assessment tools have been translated for potential use in public health, social services, forensic, and correctional settings, such as the Adolescent Sexual Abuser Project Assessment Measures (Beckett et al., 2002), and the Estimate of Risk of Adolescent Sexual Offence Recidivism (Worling and Curwen, 2001). However, since 2016, multidisciplinary practitioners and agencies have achieved more systematic competence in HSB assessment. Among the tools that have been translated and made easily accessible are those used for initial screening, such as the Sexual Behavior Continuum (Hackett, 2014) and the Traffic Light – Sexual Behavior framework (TRUE, 2015), and more comprehensive assessment tools like the earlier AIM2 and recent AIM3 (Print et al., 2009, 2012; Leonard and Hackett, 2019a,b). Although there is ample clinical experience to recommend these tools, none has been standardized or evaluated in Norwegian practice.

The ongoing nationwide AIM3 training in Norway is conducted by approved AIM3 trainers associated with the Regional Centers on Violence, Traumatic Stress and Suicide Prevention (RVTS), who use a Norwegian translation of the standardized training packet (including a competence test), developed and approved by the AIM Project in the United Kingdom. Because training in AIM3 has been carried out across agencies and a wide range of settings over the last few years, it will be useful to evaluate how this framework is understood and guides professionals in their assessment of young persons. Therefore, it is particularly relevant to explore the IRR of the AIM3 and how practitioners experience the tool and training in a Norwegian multidisciplinary context.

The research aims of this study are as follows:

1. Use a sample of multidisciplinary practitioners to estimate the IRR of the AIM3.
2. Describe the multidisciplinary practitioners’ experience of the AIM3 tool and training.

## Materials and methods

### Procedure and design

The participants in this study were recruited from a population of multidisciplinary practitioners attending one of the 15 AIM3 training courses across Norway between November 2019 and January 2022. In total, 255 practitioners attended the training courses. They were informed of the study’s nature and consented to fill out a survey and use the Norwegian version of the AIM3 assessment model (Leonard and Hackett, 2019b) to score three constructed clinical vignettes shortly after their formal training.

### Sample characteristics

A total of 56 practitioners participated in the current study. Of those, 79% were women, and 21% were men. This represents a slightly higher proportion of men than the actual share of male health and social workers in Norwegian workplaces, 16% (Norway, 2022). The participants’ mean age was 43.2 (SD 9.5, range 25–63). The sample comprised 41% psychologists, 25% social workers, and 34% other professionals (e.g., educators, nurses, and consultants) from various work settings. Their mean years of experience in professional practice was 17.6 (SD 9.5, range 0–37), and the mean years of experience working directly with children or adolescents and their families was 13.5 years (SD 9.2, range 0–31). Experience with HSB cases in the last 5 years was a mean of 4.3 cases (SD 5.0, range 0–20). The participants’ formal HSB-specific education (prior to the AIM3 training) consisted of basic HSB courses (86%) and prior AIM2 assessment training (52%); direct experience of prior assessment with the AIM2 tool was a mean of 1.3 cases (SD 2.8, range 0–17). Less than half of the sample had attended prior training courses in “AIM Intervention” (41%), in “AIM and NSPCC’s Technology-Assisted HSB assessment” (16%), and in “AIM under 12″ (13%; Carson, 2019).

### The case vignettes and survey

The case vignettes were composed to reflect a complexity and variety of known pathways and characteristics of adolescents with HSB and their caregivers, families, and networks. Some irrelevant information was included in each case to simulate real-life assessment processes. The relevant information given in each case was sufficient to score all domains or factors, but with a potential variation in score from one case to another. The case vignettes were constructed to allow the possibility of differentiated evaluations of further need for intervention and safety planning. Each case vignette’s information was limited to a maximum of four written pages, and the time was stipulated 50 to 100 min to score each with the AIM3.

The three case vignettes were based on anonymized case information drawn from prior real-life cases. The first author, with more than 30 years of clinical experience, developed and formulated the vignettes, which all center on young people having displayed various types of HSB, primarily in direct meetings with other minors. We made a deliberate exclusion in the HSB descriptions in the cases to avoid the need to supplement with the Technology-Assisted HSB assessment in scoring (Allotey and Swann, 2019a).

Participants were asked to take about 30 to 45 min to fill out the survey after scoring the case vignettes. The survey contained questions about the practitioners’ professional backgrounds; their general work experience with children, adolescents, and their families; their specific experience in assessment and HSB work; and the participants’ experiences of the AIM3 training course and in using the assessment framework or tool.

The survey and the three case vignettes used in this study were created with help from a pilot study attended by experienced HSB practitioners and trainers (*n* = 8). Their valuable feedback was used in designing the final survey and case vignettes to ensure their relevance and unambiguity (Peabody et al., 2004; Hanssen-Bauer et al., 2007; Richter and Hanssen-Bauer, 2012; Sutherland et al., 2012). The pilot group’s survey and AIM3 scores are not included in the following analysis and results.

### Measures

#### The AIM3 scores

The 25 AIM3 factors (*cf.* Table 1) were coded as follows: 4 (*significant concern*), 2 (*some concern*), or 0 (*no general concern*) or *the factor represents an area of strength* (*cf.* ordinal variables). In the AIM3, a summarized domain score (*cf.* interval variables) of 14 to 20 (in the “red band”) “may indicate an area of relative need or risk requiring specific or immediate intervention, risk management and safeguarding.” A domain score of 6 to 12 (in the “amber band”) “may indicate work is required to lower risk and meet needs requiring intervention in the medium term.” A domain score of 0 to 4 (in the “green band”) “may indicate an area of relative strength in the young person’s current presentation/context, which may be harnessed to support interventions with the young person” (Leonard & Hackett, 2019a, p. 54). The bands’ cutoffs are pragmatic and conventionally chosen, not based on psychometric analyses.

**Table 1:** An overview of the AIM3 assessment model’s domains and factors.

Domain 1: Sexual behavior	Domain 2: Nonsexual behavior	Domain 3: Developmental	Domain 4: Environmental/family	Domain 5: Self-regulation
Factor 1: Nature of the harmful sexual behavior	Factor 1: Nonsexual criminality	Factor 1: Trauma and victimization	Factor 1: Stability and safety	Factor 1: Responsibility
Factor 2: Extent of harmful sexual behavior	Factor 2: Nonsexual aggression and antisocial behavior	Factor 2: Childhood and adolescent adversity	Factor 2: Parental/carer supervision	Factor 2: Motivation and engagement
Factor 3: Victim characteristics	Factor3: Alcohol and drugs	Factor 3: Attachment	Factor 3: Relationships	Factor 3: Future perspective
Factor 4: Sexual aggression and violence	Factor 4: General behavior	Factor 4: Family functioning	Factor 4: Peer group	Factor 4: Problem-solving
Factor 5: Sexual knowledge, attitudes, and interests	Factor 5: Mental health and well-being	Factor 5: Health, intellectual, and emotional functioning	Factor 5: Education, employment and leisure	Factor 5: Social competence

Adapted from Leonard and Hackett (2019a, p. 49) with permission from the AIM project in UK.

#### Participants’ experiences of the case vignettes and the AIM3 framework/tool

The participants reported how much time they spent in the scoring process and the degree to which they experienced each case vignette as real. They were asked about the probability of their future use of the AIM3 (when relevant) and how the tool and training worked as a guide for them in making empirically informed decisions in safety and intervention planning. Further, they reported how the AIM3 tool and training affected their confidence when working with adolescents with HSB and their collaboration with multidisciplinary colleagues, as well as whether they experienced the tool as useful in evaluation of interventions and the youths’ progression. The response scales were five-point Likert scales (e.g., *not at all*, *slightly*, *neutral*, *very*, *extremely* and *very poor*, *poor*, *fair*, *good*, and *excellent*).

### Statistical analyses

In this study, we used a fully crossed ICC design in which a sample of multidisciplinary practitioners coded the same cases guided by the same assessment tool. We calculated the ICC estimates and their 95% confidence intervals using IBM SPSS statistical package version 26 (2019). These estimations were based on a two-way mixed effects model, absolute agreement, and both single and average measures reported. The single rater measurements will be applicable to a context in which a single practitioner is using the AIM3 tool, and the average measures reported will be applicable when groups of practitioners are using the AIM3.

The chosen ICC range for interpretation was “ICC values less than 0.5 are indicative of poor reliability, values between 0.5 and 0.75 indicate moderate reliability, values between 0.75 and 0.90 indicate good reliability, and values greater than 0.90 indicate excellent reliability” (Koo & Li, 2016, p. 158).

We calculated the descriptive statistics of the included study variables. In addition, we presented the categorical variables using the number of participants and percentages and the continuous variables using means and SDs.

### Ethics

The Norwegian Centre for Research Data approved this research project (reference number 626781). The participants had the opportunity to withdraw their initial consent at any time during the data collection. The first author did not train practitioners in AIM3 during the data collection period, and her own initial scores of the case vignettes and survey are not included in these analyses.

## Results

### ICC analyses of the AIM3 model

The estimated single-rater ICC for all 25 AIM3 factors (see Table 2 below) was in the moderate range (0.547; 95% *CI* [0.471, 0.634]), and the equivalent estimated average-rater ICC was in the excellent range (0.985; 95% *CI* [0.980, 0.990]). The estimated single-rater ICC for all five AIM3 domain sum scores was in the moderate range (0.697; 95% *CI* [0.548, 0.852]), and the equivalent estimated average-rater ICC was in the excellent range (0.992; 95% *CI* [0.985, 0.997]). There were no missing AIM3 data and therefore no need for missing data analysis.

**Table 2:** ICC analysis—AIM3 factor and summarized domain scores.

	Measures	Intraclass correlation coefficient (ICC)^**^	Lower bound	Upper bound	Value	df1	df2	Sig
ICC^*^ Overall AIM3 factor scores	Single	0.547	0.471	0.634	73,806	74	4,070	0.000
	Average	0.985	0.980	0.990	73,806	74	4,070	0.000
ICC Overall AIM3 domain (sum) scores	Single	0.697	0.548	0.852	157,441	14	770	0.000
	Average	0.992	0.985	0.997	157,441	14	770	0.000
Domain 1 Sexual behavior	Single	0.280	0.084	0.941	25,225	2	110	0.000
	Average	0.956	0.836	0.999	25,225	2	110	0.000
Domain 2 Nonsexual behavior	Single	0.331	0.108	0.952	38,129	2	110	0.000
	Average	0.965	0.871	0.999	38,129	2	110	0.000
Domain 3 Developmental	Single	0.785	0.489	0.993	220,469	2	110	0.000
	Average	0.995	0.982	1.000	220,469	2	110	0.000
Domain 4 Environmental/family	Single	0.804	0.517	0.994	241,548	2	110	0.000
	Average	0.996	0.984	1.000	241,548	2	110	0.000
Domain 5 Self-regulation	Single	0.729	0.412	0.991	194,190	2	110	0.000
	Average	0.993	0.975	1.000	194,190	2	110	0.000

* ICC estimates and their 95% confidence intervals were calculated by two-way mixed effects model, absolute-agreement, and single and average measures.

** “ICC values < 0.5 are indicative of poor reliability, values between 0.5 and 0.75 indicate moderate reliability, values between 0.75 and 0.90 indicate good reliability, and values > 0.90 indicate excellent reliability” (Koo & Li, 2016, p. 158).

Regarding the above reported single-rater measure for all AIM factors (0.547; 95% *CI* [0.471, 0.634]), this result indicates that 54, 7% variance of the AIM3 scores for a single practitioner is “real” (represent the construct), and that 45, 3% represent random variation. Its 95% confidence interval ranges between 0.471 and 0.634, meaning that there is 95% chance that the true ICC value lands on any point within this range. On the other hand, the equivalent average –rater measure 0.985 reported above, will indicate that 98, 5% of the variance in the mean of the whole group of practitioners is “real.” The generally higher average-measure results in our study have implications for clinical practice and will be further discussed.

For all factors (see Table 2), the estimated single-rater ICCs for Domain 1, “sexual behavior” (0.280), and for Domain 2, “nonsexual behavior” (0.331), were in the poor range. The ICC estimates for Domain 3, “developmental” (0.785), and for Domain 4, “environmental/family” (0.804), were in the good range. The ICC for Domain 5, “self-regulation,” was in the moderate range (0.729).

Supplementary Tables S1, S2 contain descriptive AIM3 scoring statistics and ICC analyses on each case separately.

### The participants’ perceptions of the three case vignettes and the AIM3 assessment

The time used for AIM3 scoring varied from 30 to 360 min. The mean time was 145 min for Case 1 (*SD* 76), 125 min for Case 2 (*SD* 60), and 106 min for Case 3 (*SD* 57). The majority of the participants (88%–100%) evaluated each of the three case vignettes as *very realistic* or *extremely realistic*. Case 1 was evaluated by 32% of the participants as *very realistic* and by 68% as *extremely realistic*, Case 2 was evaluated by 38% of participants as *very realistic* and by 61% as *extremely realistic*, and Case 3 was evaluated as *very realistic* by 43% of participants and by 45% as *extremely realistic.*

The participants reported rather high probability for future use of the AIM3 tool when relevant—30% of the participants as *very likely,* and 45% as *extremely likely*. Most of the participants perceived the AIM3 to be useful in guiding them; for example, the tool’s “empirically informed decision-making” was rated by 36% of participants as *good,* and by 59% as *excellent*. The AIM3’s usefulness in guiding “intervention planning” was rated by 52% of the participants as *good,* and by 20% as *excellent.* See Table 3 for an overview of the results from the participants’ evaluation of the AIM3 tool and training. These results have implications for clinical practice and will be further discussed.

**Table 3:** The participants’ experience of the AIM3 tool/training.

	Very poor^*^	Poor	Fair	Good	Excellent
Making empirically informed decisions	0	0	5	36	59
Safety planning	0	2	25	54	20
Intervention planning	0	2	27	52	20
Evaluation of the progression	0	2	27	46	26
Enhancing own confidence working with young persons	0	0	25	34	41
Enhancing own multidisciplinary collaboration	0	2	25	50	23

* The cells are showing % of participants (*n* = 56).

## Discussion

The aim of this study was to estimate IRR and explore how multidisciplinary practitioners in a Norwegian setting experience the AIM3 assessment model (Leonard and Hackett, 2019a,b). A reliable and consistent assessment of children and adolescents who display HSB is important for their current and future lives, their potential victims, their families, and society. Practitioners also need reliable assessment tools to help them in clinical decision-making, making less-biased judgments, and more consistently recommending interventions and risk-management measures. In light of recent years’ emergent public and professional awareness of minors who have displayed HSB, it is essential to evaluate HSB-related assessments used in the Norwegian context (Holt et al., 2016; Askeland et al., 2017). The present study’s psychometric testing of IRR also contributes to the AIM3’s more general validation process.

In this study, we found that the estimated ICC for both the factor and domain scores indicated moderate IRR for the AIM3. The majority of the raters experienced the AIM3 as, overall, of good or excellent use in guiding and helping them. Though there are no prior IRR/ICC studies on the AIM3, these overall results are reassuring for further use of the AIM3 in the Norwegian context.

### The interrater reliability of the AIM3 assessment model (Norwegian version)

The overall IRR results in this study could imply that the multidisciplinary practitioners (raters) score moderately consistently in agreement or disagreement and are moderately interchangeable when using the AIM3. The results imply that the use of the AIM3 might reduce the impact of heterogeneity of rater characteristics, experience, and competence in the field.

The term *IRR* can have different meanings and calculations (Hallgren, 2012; Evers and Sjöberg, 2013; Gwet, 2014; Hanson, 2022). One should be aware that this study’s ICC results are based on agreement in absolute terms, not only the practitioner’s relative scorings, and that the use of fewer raters, as in more traditional ICC designs, might have increased the ICC estimates further. We also wish to highlight that a moderate, better, or worse ICC absolute agreement estimate could still imply different qualitative interpretations and recommendations for intervention and safety in further case planning and follow-up (*cf.* Viljoen et al., 2019).

Comparable IRR/ICC research for other various clinical assessment tools often used in child and adolescent services in Norway (not HSB specific) has found equivalent overall ICC estimates in the moderate or good range. Examples have been given for the “HoNOSCA” (ICC 0.81; *CI* [0.70, 0.91]) and for the “Children Global Assessment Scale” (ICC = 0.61; *CI* [0.45, 0.77]; Hanssen-Bauer et al., 2007). Sutherland et al. (2012), in their study of the Risk for Sexual Violence Protocol assessment with several raters, also estimated an overall ICC in the moderate range (ICC = 0.53; *CI* [0.49, 0.56]).

The general discrepancy, in this study’s results, between the estimated ICC results for single raters (moderate) and average rater (excellent), must be emphasized and is important for the field of practice. First, ICC estimates based on average measures will generally be higher than those based on single measures (Hallgren, 2012), thus collaboration of practioners in scoring will be preferred. Second, the AIM3 factor scores were based on a three-point Likert scale (0, 2, 4) for which the mean scoring range, including the scoring disagreement average, will be limited. A more nuanced scoring range (e.g., a five-or seven-point Likert scale) might have resulted in a broader range for the agreement and disagreement mean and might have nuanced the ICC estimates based on both single and average measures. The higher ICC average measure, as compared to the single measure, recommends interrater collaboration in AIM3 scoring. This highlights the need to ensure that practitioners, especially those who must assess HSB cases alone (e.g., due to geographical reasons), receive proper and reasonable assessment consultation and support from a peer-review network. Even though the United Kingdom AIM Project and the Norwegian-approved AIM3 trainers recommend scoring based on the clinical judgment of two or more experts (consensus decision-making), clinical experience indicates great variation in scoring procedures and available support.

Regarding the overall factor scores for each domain, we see a more nuanced picture for the ICCs. Hence, estimates for Domain 3 (developmental) and Domain 4 (environmental/family) indicate good IRR; for Domain 5 (self-regulation), estimates suggest moderate IRR. However, equivalent estimated ICCs for both Domain 1 (sexual behavior) and 2 (nonsexual behavior), indicate poor IRR in this sample. This is disquieting, because Domains 1 and 2 are the most HSB-specific and unique domains in the AIM3, and they contain items commonly recognized and seen in other HSB-specific assessment tools. On the other hand, Domains 3, 4, and 5, which show good-to-moderate ICC results, include factors (items) more typical of other, more generically normed and psychometrically tested assessment tools available in Norway, such as the Achenbach Systems of Empirically Based Assessment (Achenbach, 2009) or the Behavior Assessment System for Children, Third Edition (Reynolds and Kamphaus, 2015). One apparent disadvantage with relatively comprehensive HSB assessments, such as the AIM3, is the time, effort and money spent on unnecessary, multiple, overlapping assessment for the young persons, their families, and the professionals, especially when the practitioner must assess the young person for multiple clinical problems, not only HSB. One advantage with the relatively comprehensive, ecological, holistic AIM3 framework is the collection of all 25 empirically informed factors in one place. The AIM3 ensures that the multidisciplinary practitioners do not overlook crucial HSB factors and may help the practitioners to communicate and use terms more consistently (Bloom et al., 2005). However, it is also important to note that too much data can be overwhelming for some practitioners (Munro, 2020). Another important advantage of the AIM3 is the broad assessment of HSB that it creates by taking into context multiple domains of a young person’s life and well-being, helping practitioners focus not only on the HSB itself (Campbell et al., 2020; King-Hill, 2021).

The three case vignettes used in this study have no prior “true” AIM3 scores, but in Supplementary Table S1, the mode for each of the 25 AIM3 factors on each case vignette is depicted. Supplementary Table S1 shows that all three cases are scored relatively high on the domains “sexual behavior” (D1) and “nonsexual behavior” (D2) but otherwise have more differentiated scores as intended by the case vignette’s design (*cf.* real-life assessment of complex and serious cases). The ICC results for each case vignette differ (*cf.* Supplementary Table S2). The ICC confidence interval (95%) is estimated to be in the moderate range for Case 1, the moderate-to-poor range for Case 2, and the poor range for Case 3. The attempted variation and complexity among the cases are factors that can highly influence the ICC (Bryer, 2019), but the practitioners’ endurance, time used, confidence, and sequence in the AIM3 scoring could also have some impact on these different case results.

Finally, the different AIM3 factors will vary in how difficult they are to evaluate and score in absolute agreement terms—for example, see the comparison between “Alcohol and drugs” (D2/F3) and “Sexual knowledge, attitudes, and interests” (D1/F5) or “Attachment” (D3/F3). Supplementary Table S1 shows how a few factors have no real mode score in the sample—for example, the factor “nonsexual aggression and antisocial behavior” in Case 3, “attachment” in Cases 2 and 3, and “problem-solving” in Case 2. This could mean that the information given on these factors in the relevant case vignettes is insufficient. However, the result could also imply that the AIM3 provides less guidance on these factors or that the items are, in general, difficult to score in a way that professionals can standardize.

### Participants’ evaluation of the case vignettes and the AIM3 tool/training

The majority of the multidisciplinary practitioners in the sample have validated the three case vignettes as very or extremely realistic. The result strengthens the bridging between this study and the assessment of real complex HSB cases. The moderate ICC results and time spent on the assessment process for each relative complex vignette indicate a manageable assessing time for real-life assessment practice. This is encouraging for professionals at agencies meeting the complex and serious HSB cases where thorough and broad assessments like AIM3 are adequate.

Furthermore, the results show that the majority of the participants describe their experience with the AIM3 as good or excellent when referring to helping and guiding in HSB-related case decision processing. Participants also stated that the AIM3 strengthens their confidence when meeting with adolescents and when collaborating with other multidisciplinary colleagues. The practitioners’ reported experiences are positive and indicate confidence and further use of the AIM3 in real work. However, this result can also be interpreted to mean that the practitioners’ subjective evaluations are far more positive than the limited psychometric testing indicates.

## Limitations and strengths

There are several methodological considerations that might limit the conclusions and generalizations of this study. First, out of the 255 practitioners attending formal AIM3 trainings, only 56 participated in the data collection; therefore, the results may not be representative of all practitioners with AIM3 training. The sample size (*n* = 56) is too small to investigate potential differences between groups of scorers.

The formal AIM3 trainings, the sample recruitment, and the data collection have been performed under the COVID-19 pandemic CDC guidelines. The following are the five main stated reasons for not participating in the data collection: no time for research, job change, educational leave, sickness, and extenuating family circumstances.

Second, the fully crossed design, complete AIM3 scorings, and ICC based on several multidisciplinary practitioners’ absolute agreement strengthen the validity of the study. However, the chosen form of ICC (two-way mixed effects model) will principally represent the reliability of the specific practitioners attending this study (who were not randomly sampled). Generalization to other AIM3 qualified raters, even in Norway, will be restricted.

Finally, three highly clinically based case vignettes are restricted primarily for professional and pragmatic reasons (e.g., time, recruitment, scoring endurance, and real-life resemblance versus training purpose). The case vignettes may not fully represent the complexity of HSB cases in actual outpatient settings (e.g., adolescent cases with dual “online and offline” HSB). However, the constructed case vignettes for this study are new for all the raters in the sample, which strengthens them. The strength of the raters’ agreement and disagreement could stem from the impact on the recommended intervention and social restrictions for adolescents who have displayed HSB (e.g., the ICC results impact on the AIM3 domain cutoff for the green, amber, and red bands) and further bridge the results; this possibility remains unexplored both quantitatively and qualitatively.

## Conclusion and implications

To summarize, the moderate IRR estimates and the practitioners’ generally positive experience with the AIM3 may support further use in Norwegian multidisciplinary settings. Although the Norwegian version of the AIM3 achieves moderate IRR estimates, the AIM3 book, the formal training and implementation, the use by multidisciplinary practitioners, and the research regarding anamnestic HSB assessment tools for children and adolescents must be further evaluated and developed.

The low response rate of 56 participants out of 255 attendants at the AIM3 courses also pinpoints the need for the Norwegian multisite public agencies to adjust and prepare for their employees to participate in relevant clinical and practice research. It is crucial for this strategy to be developed and claimed for children and adolescents who display HSB, not only for theoretically based, empirically informed studies but also for empirically tested and evaluated assessment practices.

The implications of this exploratory study indicate that more research is needed to evaluate and optimize the HSB-specific factors, here included in the AIM3 (e.g., Domains 1 and 2), and further evaluate how this could correspond to similar factors in other HSB related tools. Furthermore, there are a few relevant questions related to the formal AIM3 trainings that must be explored and tested. For example, would it be more useful for the training participants to score the AIM3 competence test in dual collaboration (*cf.* better average measures) during or after the formal time-restricted AIM3 trainings?

Further implications of this study also indicate the need for more research (including multimethod and qualitative methods) that focuses on the ways practitioners bridge the decision processes from AIM3 assessment scores with the interpretation to real-life follow-up of children and adolescents who have displayed HSB. Could the formal trainings for the AIM3 and the examples provided from AIM Intervention Guidance be integrated more? Recommending too much or too little intervention, restrictions, and restraints may be unethical and even harmful to minors in their ongoing development and maturation. Information, such as the ways children and adolescents report their own experiences during assessment and intervention, will be important (Campbell et al., 2020).

This study provides some input on the future evaluation, validation, and development of the original AIM3 and for other HSB related assessment tools. Despite methodological limitations, this study implies how important it is in general for the developers of assessment tools to perform initial research (e.g., psychometrics) before implementing and further evaluating their products (Evers and Sjöberg, 2013; Nøkleby et al., 2020; Helland et al., 2022). In general, just changing “the name and the game” of a tool, for example (for more actuary risk prediction to a more anamnestic and responsiveness assessment purpose), should not be reassuring enough for adolescents, HSB victims, society, or professional users and employers. The responsibility of the developers and authors should be transparent; moreover, it is essential that international and national public agencies and professionals who teach or use the different tools in practice accept responsibility. Health and social workers, just like agencies and charities that recommend available HSB-related tools, should be aware of, and transparent about, the tools’ strengths and limitations (e.g., the purpose, population, context, reliability, and validity).

## Data availability statement

The raw data supporting the conclusions of this article will be made available by the authors, without undue reservation.

## Ethics statement

The studies involving human participants were reviewed and approved by the Norwegian Centre for Research Data approved this research project (reference number 626781). The patients/participants provided their written informed consent to participate in this study.

## Author contributions

All authors listed have made a substantial, direct, and intellectual contribution to the work and approved it for publication.

## Conflict of interest

The authors declare that the research was conducted in the absence of any commercial or financial relationships that could be construed as a potential conflict of interest.

## Publisher’s note

All claims expressed in this article are solely those of the authors and do not necessarily represent those of their affiliated organizations, or those of the publisher, the editors and the reviewers. Any product that may be evaluated in this article, or claim that may be made by its manufacturer, is not guaranteed or endorsed by the publisher.

## Supplementary material

The Supplementary material for this article can be found online at: https://www.frontiersin.org/articles/10.3389/fpsyg.2022.1019739/full#supplementary-material
