# Supplementary material for: Interrater reliability and experiences of Assessment, Intervention, and Moving-on 3 Assessment Model in a multidisciplinary Norwegian sample
Source: Front Psychol. 2022 Dec 5;13:1019739. doi: 10.3389/fpsyg.2022.1019739 (PMC9762422; doi:10.3389/fpsyg.2022.1019739)
Supplement: Supplementary file 1 [file Data_Sheet_1.docx]

**Supplemental Material**

**Table S1**

*Descriptive Statistics — AIM3 Factor Scorings on Each Case*

| AIM3  Domain (D)  Factor (F) | AIM3 scores | Case 1  **Mode** | Case 2  **Mode** | Case 3  **Mode** |
| --- | --- | --- | --- | --- |
| **Domain 1**  **Sexual Behavior** |  | % | % | % |
| F1 Nature of Harmful Sexual Behavior | 0 | 1.8 | 1.8 | 1.8 |
|  | 2 | 32.1 | 12.5 | **50.0** |
|  | 4 | **66.1** | **85.7** | 48.2 |
| F2 Extent of Harmful Sexual Behavior | 0 | 7.1 | 3.6 | 7.1 |
|  | 2 | **60.7** | 33.9 | **80.4** |
|  | 4 | 32.1 | **62.5** | 12.5 |
| F3 Victim Characteristics | 0 | 14.3 | 0 | 1.8 |
|  | 2 | **78.6** | 7.1 | **66.1** |
|  | 4 | 7.1 | **92.9** | 32.1 |
| F4 Sexual Aggression and Violence | 0 | 1.8 | 23.2 | **57.1** |
|  | 2 | **57.1** | **60.7** | 42.9 |
|  | 4 | 41.1 | 16.1 | 0 |
| F5 Sexual Knowledge, Attitudes, and Interests | 0 | 5.4 | 3.6 | 7.1 |
|  | 2 | **64.3** | **57.1** | **58.9** |
|  | 4 | 30.4 | 39.3 | 33.9 |
| **Domain 2**  **Nonsexual Behavior** |  |  |  |  |
| F1 Nonsexual Criminality | 0 | **96.4** | **55.4** | 30.4 |
|  | 2 | 3.6 | 41.1 | **42.9** |
|  | 4 | 0 | 3.6 | 26.8 |
| F2 Nonsexual Aggression and Antisocial Behavior | 0 | **96.4** | 21.4 | 48.2 |
|  | 2 | 1.8 | **60.7** | 48.2 |
|  | 4 | 1.8 | 17.9 | 3.6 |
| F3 Alcohol and Drugs | 0 | 30.4 | **100** | **92.9** |
|  | 2 | **64.3** | 0 | 7.1 |
|  | 4 | 5.4 | 0 | 0 |
| F4 General Behavior | 0 | **89.3** | 28.6 | 16.1 |
|  | 2 | 10.7 | **60.7** | **62.5** |
|  | 4 | 0 | 10.7 | 21.4 |
| F5 Mental Health and Well-Being | 0 | 30.4 | 0 | **51.8** |
|  | 2 | **64.3** | **58.9** | 39.3 |
|  | 4 | 5.4 | 41.1 | 8.9 |
| **Domain 3**  **Developmental** |  |  |  |  |
| F1 Trauma and Victimization | 0 | **71.4** | 0 | **57.1** |
|  | 2 | 26.8 | 10.7 | 30.4 |
|  | 4 | 1.8 | **89.3** | 12.5 |
| F2 Childhood and Adolescent Adversity | 0 | 5.4 | 0 | 10.7 |
|  | 2 | **87.5** | 5.4 | **64.3** |
|  | 4 | 7.1 | **94.6** | 25.0 |
| F3 Attachment | 0 | 7.1 | 3.6 | 42.9 |
|  | 2 | **85.7** | 48.2 | 42.9 |
|  | 4 | 7.1 | 48.2 | 14.3 |
| F4 Family Functioning | 0 | 8.9 | 1.8 | **78.6** |
|  | 2 | **89.3** | 39.3 | 17.9 |
|  | 4 | 1.8 | **58.9** | 3.6 |
| F5 Health, Intellectual, and Emotional Functioning | 0 | 37.5 | 0 | 32.1 |
|  | 2 | **60.7** | 33.9 | **60.7** |
|  | 4 | 1.8 | **66.1** | 7.1 |
| **Domain 4**  **Environmental/Family** |  |  |  |  |
| F1 Stability and Safety | 0 | **80.4** | 1.8 | 10.7 |
|  | 2 | 19.6 | 41.1 | **62.5** |
|  | 4 | 0 | **57.1** | 26.8 |
| F2 Parental/Carer Supervision | 0 | 3.6 | 0 | **42.9** |
|  | 2 | **69.6** | 28.6 | 41.1 |
|  | 4 | 26.8 | **71.4** | 16.1 |
| F3 Relationships | 0 | **67.9** | 3.6 | **83.9** |
|  | 2 | 32.1 | **57.1** | 14.3 |
|  | 4 | 0 | 39.3 | 1.8 |
| F4 Peer Group | 0 | **69.6** | 8.9 | **78.6** |
|  | 2 | 28.6 | 37.5 | 19.6 |
|  | 4 | 1.8 | **53.6** | 1.8 |
| F5 Education, Employment, and Leisure | 0 | **98.2** | 3.6 | **94.6** |
|  | 2 | 1.8 | **66.1** | 3.6 |
|  | 4 | 0 | 30.4 | 1.8 |
| **Domain 5**  **Self-Regulation** |  |  |  |  |
| F1 Responsibility | 0 | 0 | 0 | 30.9 |
|  | 2 | 23.2 | 23.2 | **61.8** |
|  | 4 | **76.8** | **76.8** | 7.3 |
| F2 Motivation  and Engagement | 0 | 12.5 | 23.2 | **78.6** |
|  | 2 | **75.0** | **60.7** | 21.4 |
|  | 4 | 12.5 | 16.1 | 0 |
| F3 Future Perspective | 0 | **91.1** | 7.1 | **89.3** |
|  | 2 | 8.9 | **57.1** | 10.7 |
|  | 4 | 0 | 35.7 | 0 |
| F4 Problem-Solving | 0 | 44.6 | 7.1 | **55.4** |
|  | 2 | **48.2** | 46.4 | 39.3 |
|  | 4 | 7.1 | 46.4 | 5.4 |
| F5 Social Competence | 0 | **58.9** | 1.8 | **67.9** |
|  | 2 | 39.3 | 46.4 | 30.4 |
|  | 4 | 1.8 | **51.8** | 1.8 |

**Table S2**

*ICC Estimated for Each Case Vignette*

S2.1 ICC Estimated for Case 1

|  |  | Measures | ICC* | Lower Bound | Upper Bound | Value | Df1 | Df2 |
| --- | --- | --- | --- | --- | --- | --- | --- | --- |
| ICC for All  Factors (25) |  | Single | .556 | .439 | .718 | 82.780 | 24 | 1320 |
|  |  | Average | .986 | .978 | .993 | 82.780 | 24 | 1320 |
| Sum Scores | Mode | Mean | SD |  |  |  |  |  |
| D1 Sexual Behavior | 16 | 12.9 | 3.50 |  |  |  |  |  |
| D2 Nonsexual Behavior | 2 | 3.4 | 2.10 |  |  |  |  |  |
| D3 Developmental | 8 | 7.8 | 2.28 |  |  |  |  |  |
| D4 Environmental/ Family | 4 | 4.2 | 1.99 |  |  |  |  |  |
| D5  Self-Regulation | 8 | 7.8 | 2.54 |  |  |  |  |  |

S2.2 ICC Estimated for Case 2

|  |  | Measures | ICC* | Lower Bound | Upper Bound | Value | Df1 | Df2 |
| --- | --- | --- | --- | --- | --- | --- | --- | --- |
| ICC for All  Factors (25) |  | Single | .442 | .320 | .608 | 52.760 | 24 | 1320 |
|  |  | Average | .978 | .964 | .989 | 52.760 | 24 | 1320 |
| Sum Scores | Mode | Mean | SD |  |  |  |  |  |
| D1 Sexual Behavior | 16 | 15.1 | 3.00 |  |  |  |  |  |
| D2 Nonsexual Behavior | 8 | 7.4 | 3.00 |  |  |  |  |  |
| D3 Developmental | 18 | 17.0 | 2.41 |  |  |  |  |  |
| D4 Environmental/ Family | 14 | 14.7 | 3.44 |  |  |  |  |  |
| D5  Self-Regulation | 14 | 13.8 | 3.33 |  |  |  |  |  |

S2.3 ICC Estimated for Case 3

|  |  | Measures | ICC* | Lower Bound | Upper Bound | Value | Df1 | Df2 |
| --- | --- | --- | --- | --- | --- | --- | --- | --- |
| ICC for All Factors (25) |  | Single | .365 | .254 | .531 | 39.996 | 24 | 1320 |
|  |  | Average | .970 | .950 | .984 | 39.996 | 24 | 1320 |
| Sum scores | Mode | Mean | SD |  |  |  |  |  |
| D1 Sexual Behavior | 10 | 11.0 | 3.10 |  |  |  |  |  |
| D2 Nonsexual Behavior | 6 | 6.4 | 3.49 |  |  |  |  |  |
| D3 Developmental | 4 | 6.8 | 3.87 |  |  |  |  |  |
| D4 Environmental/ Family | 4 | 4.8 | 3.13 |  |  |  |  |  |
| D5  Self-Regulation | 2 | 3.9 | 3.17 |  |  |  |  |  |

*Note.* (*) ICC estimates and their 95% confidence intervals were calculated by two-way mixed effects model, absolute-agreement, and single and average measures.
